# Neurovascular Interaction Promotes the Morphological and Functional Maturation of Cortical Neurons

**DOI:** 10.3389/fncel.2017.00290

**Published:** 2017-09-15

**Authors:** Kun-Wei Wu, Jia-Lin Mo, Zeng-Wei Kou, Qi Liu, Ling-Ling Lv, Yu Lei, Feng-Yan Sun

**Affiliations:** ^1^Institute of Biomedical Sciences and Department of Neurobiology, School of Basic Medical Sciences, Shanghai Medical College, Fudan University Shanghai, China; ^2^Shanghai Key Laboratory of Clinical Geriatric Medicine, Research Center on Aging and Medicine, Shanghai Medical College, Fudan University Shanghai, China; ^3^State Key Laboratory of Medical Neurobiology, Fudan University Shanghai, China

**Keywords:** neurovascular unit, brain development, co-culture, synaptic function, VEGF, p38 MAPK

## Abstract

Brain microvascular endothelial cells (BMEC) have been found to guide the migration, promote the survival and regulate the differentiation of neural cells. However, whether BMEC promote development and maturation of immature neurons is still unknown. Therefore, in this study, we used a direct endothelium-neuron co-culture system combined with patch clamp recordings and confocal imaging analysis, to investigate the effects of endothelial cells on neuronal morphology and function during development. We found that endothelial cells co-culture or BMEC-conditioned medium (B-CM) promoted neurite outgrowth and spine formation, accelerated electrophysiological development and enhanced synapse function. Moreover, B-CM treatment induced vascular endothelial growth factor (VEGF) expression and p38 phosphorylation in the cortical neurons. Through pharmacological analysis, we found that incubation with SU1498, an inhibitor of VEGF receptor, abolished B-CM-induced *p*-p38 upregulation and suppressed the enhancement of synapse formation and transmission. SB203580, an inhibitor of p38 MAPK also blocked B-CM-mediated synaptic regulation. Together these results clearly reveal that the endothelium-neuron interactions promote morphological and functional maturation of neurons. In addition, neurovascular interaction-mediated promotion of neural network maturation relies on activation of VEGF/Flk-1/p38 MAPK signaling. This study provides novel aspects of endothelium-neuron interactions and novel mechanism of neurovascular crosstalk.

## Introduction

It has been well known that neurovascular interaction plays important roles in the development of brain architecture and its function in the mammalian (Iadecola, 2004; Stubbs et al., 2009; Eichmann and Thomas, 2013; Andreone et al., 2015). During development, neurovascular co-patterning mediates cell fate determination and navigates towards their targets in central nervous system (CNS) and peripheral nervous system. It has been reported that CNS-derived axonal guidance molecules, such as slits, semaphorins, ephrins and netrins, also guide the blood vessel outgrowth (Autiero et al., 2005; Tam and Watts, 2010) and vascular-derived signals, such as artemin and endothelin, involve in axonal guidance (Honma et al., 2002; Makita et al., 2008). In addition, local neural activity affects vascular branching and density in the cortex (Lacoste et al., 2014; Whiteus et al., 2014) and cerebral vascularization impairment inhibits neuronal proliferation (Haigh et al., 2003; Raab et al., 2004). Recent studies illustrate the molecular and cellular mechanism of neurovascular interaction in the neural development with different co-culture models. Shen et al. (2004) with an indirect transwell co-culture system, have found that endothelial cells stimulate the self-renewal of embryonic and adult neural stem cells via activating Notch and Hes1 and enhance their neuron generation. Through direct cell contact, endothelial cells potentiate neuronal differentiation of embryonic neural stem cells in a direct co-culture system (Gama Sosa et al., 2007). Moreover, it has been reported that endothelial cells enhance neurogenesis and migration of neurons in co-cultured subependymal zone explants (Leventhal et al., 1999) and accelerate neurite outgrowth of newborn neurons in co-cultured spinal motor neurons (Dugas et al., 2008). These observations suggest that neural and vascular cells form a functionally integrated network in the developing CNS. It is not known, however, whether brain endothelial cells promote maturation of neurons and regulate function of brain neural network during postnatal development under physiological conditions.

Brain microvascular endothelial cells (BMEC) have been found to produce and release a wide variety of molecules, such as brain-derived neurotrophic factor (Kim et al., 2004; Guo et al., 2008), vascular endothelial growth factor (VEGF; Li et al., 2006; He et al., 2016) and nitric oxide (Katusic and Austin, 2014). These factors are known to regulate neuronal activity (Bolton et al., 2000; Ruiz de Almodovar et al., 2009; Wu et al., 2016) which raises the possibility that endothelial cells modulate maturation of neuronal network via producing and releasing the soluble factors in the developing CNS. VEGF, as a classical angiogenic molecule, also participate in neurogenesis (Jin et al., 2002; Wang et al., 2007; Shen et al., 2016), neuronal migration (Ruiz de Almodovar et al., 2010) and dendritogenesis (Rosenstein et al., 2003; Wang et al., 2009). In the developing brain, VEGF expression by neural cells as a paracrine factor for endothelial cells induces brain angiogenesis and guides the vessel growth. However, in turn, whether and how VEGF acts as a paracrine or autocrine factor for neurons to regulate the neuronal maturation and activity remains elusive.

In this study, we investigated the regulatory effects of endothelial cells on morphological and functional maturation of developing neurons. With a direct co-culture model combined with electrophysiological recordings and confocal imaging analysis, we discovered that brain endothelial cells directly promoted neurite outgrowth of cortical neurons in the early stage of *in vitro* development and enhanced synapse formation and transmission via VEGF/Flk-1/p38 MAPK signaling in the later stage of *in vitro* development. Our results provide novel aspects of neurovascular interaction mediated by VEGF on the modulation of neural network construction during development.

## Materials and Methods

### Preparation of Primary Cortical Neurons and Brain Microvascular Endothelial Cells Cultures

Cortical neurons were obtained from 16-day-old C57BL/6 mice embryos as described previously (Wu et al., 2016). In brief, after removal of blood vessels and pia mater, the dissected cerebral cortices were digested with trypsin. Following digestion, the precipitate was re-suspended and the isolated cells were plated at a density of 2 × 10^5^ cells/ml on poly-L-lysine-coated dishes. Cells were grown with the medium based on Neurobasal medium containing 2% B27 supplement in a 37°C and 5% CO_2_ incubator. Half the volume of the medium was changed every 3 days.

BMEC were obtained from 2-day-old neonatal C57BL/6 mice as described previously (Wu et al., 2016). In brief, after removal of brainstems, cerebella and thalami, the isolated forebrain was digested with type-2 collagenase. After digestion, the precipitate was re-suspended in 20% BSA and centrifuged. Re-suspend the bottom cells, carefully add on the top of the 50% Percoll gradient and centrifuge again. The microvessel endothelial cells were collected and plated on dishes pre-coated with gelatin. Cells were grown with EGM-2 medium in a 37°C and 5% CO_2_ incubator. The culture medium was changed every 3 days.

### Preparation of the Direct Endothelium-Neuron Co-Culture and Brain Microvascular Endothelial Cells-Conditioned Medium

BMEC were trypsinized and collected via centrifugation; then the cells were re-suspended in Neurobasal containing 2% B27 supplement, counted and added to 1 or 12 days *in vitro* (DIV) neuronal cultures at a density of 2 × 10^4^ cells/ml to establish the direct co-culture model.

After the endothelial cells grew to 80% confluency, the cultured medium was replaced with Neurobasal supplemented with 2% B27 and harvested after 6 h as BMEC-conditioned medium (B-CM). The conditioned medium was passed through a 0.22 μm filter before use. For B-CM treatment, half the volume of the medium in the neuron cultures was changed with mixed medium of Neurobasal containing 2% B27 supplement and B-CM (1:1). Neurons were co-cultured with BMEC or treated with B-CM for 2 days and then the neuronal morphology and electrophysiological activities were evaluated.

For the inhibitors experiments, the neurons were treated with B-CM in the presence or absence of the Flk-1 inhibitor SU1498 (10 μM, Abcam, Cambridge, UK) and p38 inhibitor SB203580 (10 μM, SelleckChem, Houston, TX, USA).

### Patch-Clamp Recordings

Cortical pyramidal neurons identified by their characteristic morphology were selected for electrophysiological recordings. Whole-cell currents were recorded using electrodes pulled from borosilicate glass capillaries on a puller (P-97, Sutter Instrument Co., Novato, CA, USA). Patch electrodes had an open-tip resistance of 6–10 MΩ when filled with intracellular solution (mM): K gluconate 150, MgSO_4_·7H_2_O 2, CaCl_2_ 0.1, EGTA 1, K_2_ATP 2, Na_3_GTP 0.1, HEPES 10, pH was adjusted to 7.4 with KOH.

Resting membrane potential (RMP) and membrane capacitance (Cm) were measured after the whole-cell configuration. The input resistance (R_in_) was calculated from the plot of current vs. voltage. Action potentials (APs) were evoked by a series of 400 ms depolarizing current stimulations (from −400 pA to +300 pA with 50 pA steps) in current-clamp configuration. Spontaneous miniature excitatory postsynaptic currents (mEPSC) were recorded in the individual pyramidal neurons held at –65 mV in voltage-clamp configuration in the presence of TTX (1 μM, Sigma-Aldrich, Louis, MO, USA) and picrotoxin (10 μM, Sigma-Aldrich) in the extracellular solution to block the generation and propagation of spontaneous APs and inhibitory postsynaptic currents.

Signals were filtered at 2 kHz and sampled at 10 kHz using AXOPATCH 700B amplifier (Axon Instruments, Foster City, CA, USA), DigiData 1322A interface (Axon Instruments) and Clampex 10.0 software (Axon Instruments). All electrophysiological data were analyzed off-line using the software Clampfit 10.0 (Axon Instruments) or Mini Analysis Program (Synaptosoft, Decatur, GA, USA).

### Western Blot

Western blotting was carried out on the cell lysates or culture medium. Cell-free conditioned medium was collected and concentrated using ultracentrifugation filter devices (3-kDa cut-off, Millipore, Billerica, MA, USA). Proteins were separated by SDS-polyacrylamide gel electrophoresis, transferred onto polyvinylidene difluoride membranes, and incubated with primary antibodies against VEGF (1:500; catalog sc-152; Santa Cruz Biotechnology, Santa Cruz, CA, USA), p38 (1:1000; catalog 9212; Cell Signaling Technology, Beverly, MA, USA) and phosphorylated p38 (*p*-p38, 1:1000; catalog 4511; Cell Signaling Technology), followed by incubation with horseradish peroxidase (HRP)-conjugated secondary antibody (1:2000; catalog sc-2004; Santa Cruz Biotechnology). The immunoreactive proteins were visualized by using enhanced chemiluminescence detection kit (Santa Cruz Biotechnology) and ImageQuant LAS 4000 imager (GE Healthcare, Abingdon, UK). Optical density was assessed using ImageJ. All experiments were performed at least three times.

### Immunocytochemistry

Neurons on 1 DIV were co-cultured with BMEC or treated with B-CM. After 2 days, neurons were fixed and stained with mouse monoclonal antibody MAP2 (1:200; catalog M4403; Sigma-Aldrich) and then incubated with anti-mouse IgG Alexa Fluor 594-conjugated secondary antibody (1:1000; catalog 21203; Invitrogen, Carlsbad, CA, USA).

To test the effects of BMEC co-culture or B-CM on synaptogenesis, neurons on 12 DIV were co-cultured with BMEC or treated with B-CM for 2 days. On 14 DIV, neurons were fixed and stained for presynaptic and postsynaptic markers VGlut-1 (rabbit anti-VGlut-1, 1:200; catalog 48-2400; Invitrogen) and PSD95 (mouse anti-PSD95, 1:200; catalog MAB1596; Millipore). Alexa-conjugated secondary antibodies (anti-rabbit IgG Alexa Fluor 594, 1:1000; catalog A21207 and anti-mouse IgG Alexa Fluor 488, 1:1000; catalog A21202) were used for detection. The fluorescent images were captured using a confocal microscope (SP8, Leica, Wetzlar, Germany).

### Cell Viability Analysis

For CCK8 assays, cells were plated in a 96-well plate, processed according to the manufacturer’s instructions (CCK8, Dojindo, Kumamoto, Japan) and measure the absorbance at 450 nm using a microplate reader (Thermos scientific, Fremont, CA, USA).

### Neurite, Spine and Synapse Analysis

For neurite analysis, cortical neurons on 3 DIV were stained for MAP2 and those on 14 DIV were filled individually with Lucifer yellow (4.2 mM, Sigma-Aldrich) using the electrodes for 3 min, and fixed in paraformaldehyde. Images were captured with the confocal microscope (SP8, Leica). The total length of neurites were quantified using ImageJ with the NeuronJ plugin. The neuronal complexity was analyzed by Sholl analysis, which measures the number of intersections of the dendrites crossing a series of concentric circles from the soma using ImageJ with the Sholl analysis plugin.

Dendritic spine of the cortical pyramidal neurons on 14 DIV was visualized by Lucifer-Yellow label and counted using ImageJ. An average of two individual dendritic segments (20–70 μm each) per neuron was randomly chosen for spine analysis.

Synaptic cluster densities were determined by counting the number of VGlut-1, PSD95 and co-localized puncta per 10 μm dendrite in cortical pyramidal neurons using Image-Pro Plus 6.0 software (Media Cybernetics, Rockville, MD, USA). For this analysis, an average of 2 seperate proximal dendritic segments (16–60 μm each; distance from the soma >10 μm) per neuron was randomly chosen.

### Statistical Analysis

All data were analyzed using Stata, version 12 (StataCorp, College Station, TX, USA) and statistical significance was determined using chi-square test for percentages, Kolmogorov–Smirnov test for cumulative probabilities distribution, Student’s *t*-test for comparison of two groups, or ANOVA and Tukey *post hoc* test for comparison of multiple groups. For the parametric test, the normality of each data set was verified by Shapiro-Wilk W test. Statistical differences were defined as *p* < 0.05.

## Results

### BMEC Regulate Neurite Outgrowth and Spine Formation of Cortical Neurons

**Figure 1:**
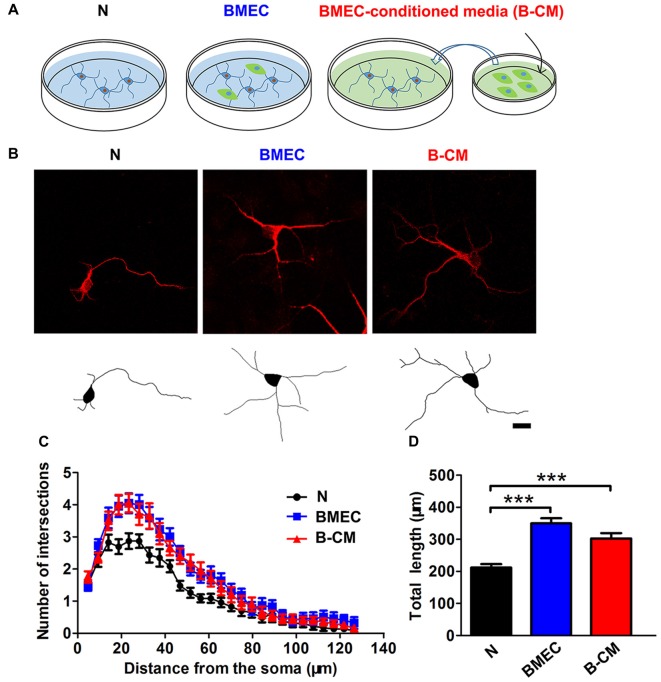
Brain microvascular endothelial cells (BMEC) promote neurite outgrowth during early neuronal development. **(A)** Schematic representation of experimental design. Neurons were co-cultured with BMEC or treated with BMEC-conditioned medium (B-CM) for 2 days. **(B)** Representative confocal images (*upper*) and skeletonized traces (*lower*) of neurons grown with BMEC or treated with B-CM for 2 days compared with neurons cultured alone (N) at days *in vitro* (DIV 3). Scale bar = 20 μm.** (C)** The neurite complexity is shown by Sholl analysis.** (D)** The quantification of total neurite length is shown in the bar graph (N: *n* = 23; BMEC: *n* = 23; B-CM: *n* = 20. ***p < 0.001).

To investigate whether BMEC regulated neurite outgrowth, cortical neurons on 1 DIV were co-cultured with BMEC or treated with B-CM for 2 days and then stained for MAP2. We found that BMEC co-culture or B-CM treatment increased neurite length and complexity, compared with neurons cultured alone in the early stage (1–3 DIV) of *in vitro* development (Figure 1).

**Figure 2:**
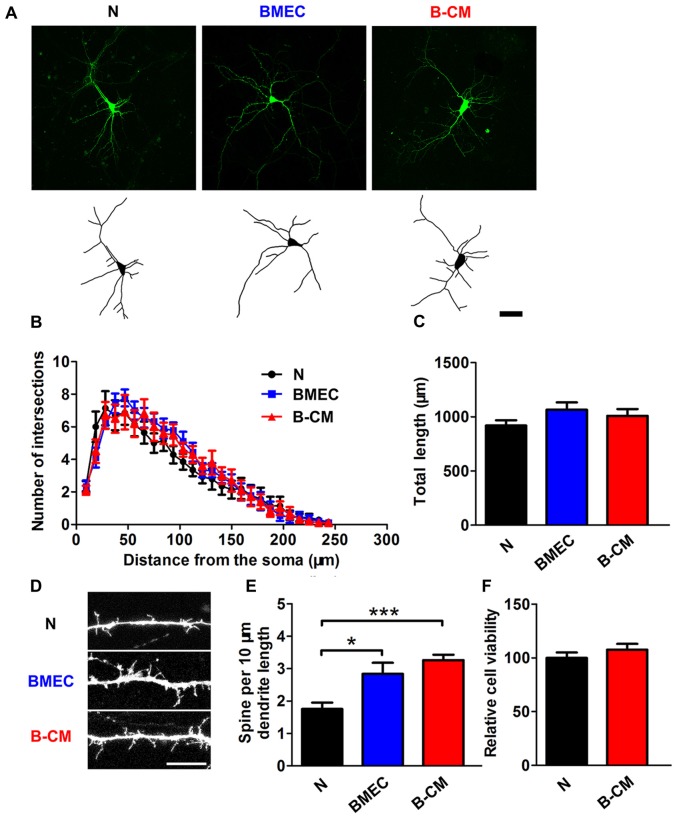
BMEC promote spine formation in cortical neurons. **(A)** Representative confocal images (*upper*) and skeletonized traces (*lower*) of neurons grown with BMEC or treated with B-CM compared with neurons cultured alone (N) at DIV 14. Scale bar = 50 μm. **(B)** The dendrite complexity is shown by Sholl analysis. **(C)** The quantification of total dendrite length is shown in the bar graph (N: *n* = 14; BMEC: *n* = 9; B-CM: *n* = 10). **(D)** The representative image of a dendritic segment from neurons cultured alone, co-cultured with BMEC or treated with B-CM. Scale bar = 5 μm. **(E)** The quantification of spine density in control and treated neurons is shown (N: *n* = 8; BMEC: *n* = 7; B-CM: *n* = 9. *p < 0.05; ***p < 0.001).** (F)** Cell viability of neurons cultured alone or treated with B-CM is shown in the bar graph (data from three independent experiments).

We next evaluated the effect of endothelial cells on dendritic morphology in the later stage (12–14 DIV) of *in vitro* development. At this age, neurons are nearly mature and early synaptic contacts and dendritic spines form (Ron et al., 2012). By labeling dendrites with Lucifer-Yellow, we found that there was no significant changes of dendrite length and complexity of neurons co-cultured with BMEC or treated with B-CM comparing with those of neurons cultured alone (Figures 2A–C), whereas we observed a significant increase in dendritic spine density in the treated neurons (Figures 2D,E). These observations suggest that BMEC and secreted factors promote cortical neurite outgrowth in the early stage of *in vitro* development and accelerate dendritic spine formation in the later stage of *in vitro* development. Additionally, to assess whether these effects of endothelial cells on neuronal morphology were the result of changes in cell viability, we carried out CCK8 assays in neurons cultured alone and B-CM-treated neurons. As shown in Figure 2F, B-CM treatment did not affect neuronal viability.

### BMEC Promote Electrophysiological Development of Cortical Neurons

**Figure 3:**
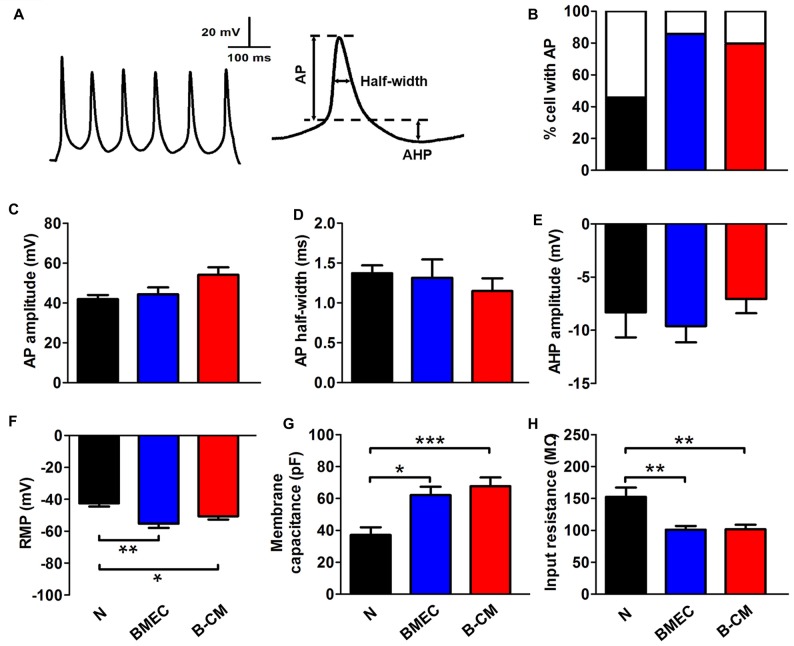
BMEC promote electrophysiological development in cortical neurons. **(A)** The typical whole-cell current clamp recordings with depolarizing current (50 pA) injection in cortical neurons co-cultured with BMEC for 2 days. Scale bar is shown in the top. The right panel represents action potential (AP) measures taken. **(B)** Summarized data show that significantly more neurons display AP spikes when grown with BMEC or treated with B-CM than those cultured alone (N) (N: *n* = 24; BMEC: *n* = 21; B-CM: *n* = 24. N vs. BMEC, *p* < 0.05; N vs. B-CM, *p* < 0.05). **(C–E)** The panels represent the measurements taken for AP amplitude, AP half-width and after hyperpolarization (AHP) amplitude respectively. There is no significant difference of AP properties among three groups (N: *n* = 9; BMEC: *n* = 16; B-CM: *n* = 14). **(F–H)** Summary graphs showing the passive membrane properties (Resting membrane potential (RMP), membrane capacitance (Cm) and input resistance (R_in_)) of cortical neurons grown alone, co-cultured with BMEC or treated with B-CM (*n* = 10 per condition. *p < 0.05; **p < 0.01; ***p < 0.001).

We further investigated whether these effects on the morphology of neurons induced by endothelial cells had functional consequences at the electrophysiological level. For this purpose, we first recorded APs by stimulating neurons with a series of 400 ms depolarizing current in control and treated neurons. We found that the percentage of success to induce APs in the co-culture neurons (85.71%) or B-CM-treated neurons (79.70%) was increased, compared with control neurons (45.83%; Figure 3B), indicating that the stimulations are more efficient to produce APs of neurons in the co-culture or B-CM treatment conditions. Additionally, the characteristics of the APs (amplitude, half-width and after hyperpolarization) recorded from co-culture neurons and B-CM-treated neurons were similar to those cultured alone (Figures 3C–E), indicating that BMEC and secreted factors promote neuronal APs production but not further maturation. We next analyzed the passive membrane properties (RMP, Cm and R_in_) and found that BMEC co-culture or B-CM treatment induced the developmental changes in them (Figures 3F–H).

### BMEC Increase mEPSC Frequency of Cortical Neurons

**Figure 4:**
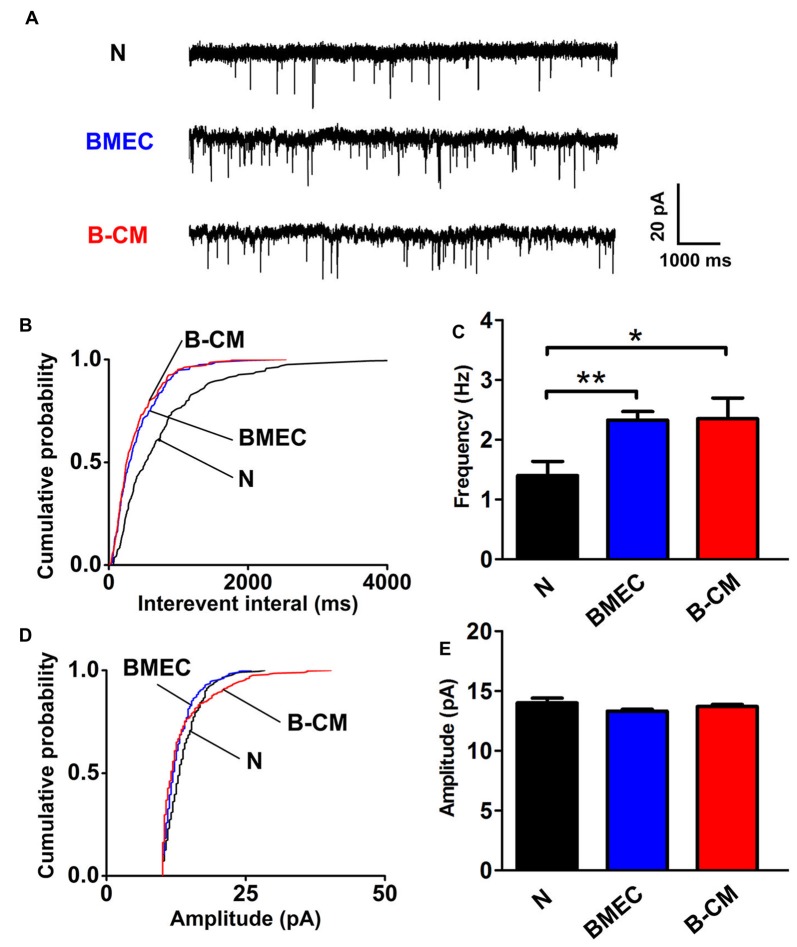
BMEC increase miniature excitatory postsynaptic currents (mEPSC) frequency. **(A)** Example traces from neurons cultured alone, co-cultured with BMEC or treated with B-CM showing mEPSC recorded in the presence of 1 μM TTX and 10 μM picrotoxin at −65 mV. Scale bar is shown in the right. **(B,C)** Cumulative probability plots of interevent interval and the quantification of mEPSC frequency are shown. **(D,E)** Cumulative probability plot of mEPSC amplitude and the quantification of mEPSC amplitude are shown (*n* = 13 per condition. *p < 0.05; **p < 0.01).

The enhanced electrophysiological development of cortical neurons always parallels with an increase in the synaptic transmission and for this reason, we evaluated mEPSC of cortical neurons. Using whole-cell patch-clamp, mEPSC were recorded from voltage-clamped cortical pyramidal neurons held at –65 mV in the presence of TTX and picrotoxin. We found that BMEC co-culture or B-CM treatment significantly decreased the interevent interval between mEPSC (increased frequency) with no changes of mEPSC amplitude in cortical neurons (Figure 4).

### SU1498 and SB203580 Inhibit the Increase of mEPSC Frequency of Cortical Neurons Induced by B-CM

**Figure 5:**
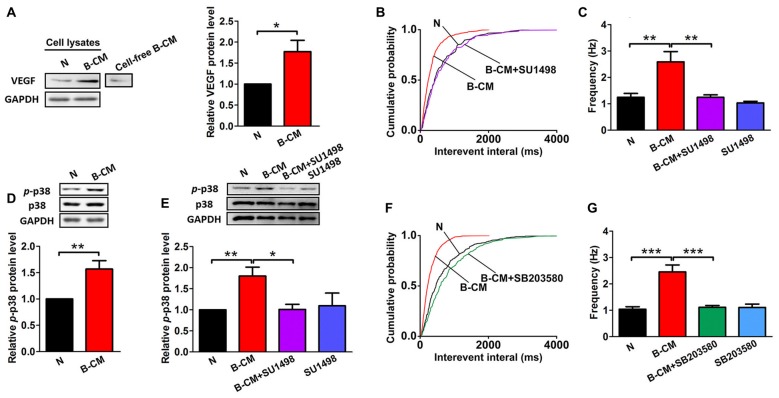
SU1498 and SB203580 inhibit the increase of mEPSC frequency induced by B-CM. **(A)** Representative blots for vascular endothelial growth factor (VEGF) and GAPDH in neurons cultured alone (N) or treated with B-CM for 2 days as well as VEGF in cell-free B-CM. The relative quantity of VEGF protein was calculated after normalization to GAPDH (data from three independent experiments. *p < 0.05). **(B,C)** Cumulative probability plots of interevent interval and the quantification of mEPSC frequency are shown (N: *n* = 7; B-CM: *n* = 10; B-CM+SU1498: *n* = 8; SU1498: *n* = 10. **p < 0.01). **(D)** Representative blots for *p*-p38, p38 and GAPDH in neurons cultured alone or treated with B-CM for 2 days. The relative quantity of *p*-p38 protein was calculated after normalization to GAPDH (data from seven independent experiments. **p < 0.01). **(E)** Representative blots for *p*-p38, p38 and GAPDH in neurons cultured alone or treated with SU1498, B-CM or both. The relative quantity of *p*-p38 protein was calculated after normalization to GAPDH (data from four independent experiments. *p < 0.05; **p < 0.01). **(F,G)** Cumulative probability plot of interevent interval and the quantification of mEPSC frequency are shown (N: *n* = 10; B-CM: *n* = 10; B-CM+SB203580: *n* = 8; SB203580: *n* = 7. ***p < 0.001). Details see Supplementary Figure S1.

Given our findings that BMEC co-culture and B-CM have similar regulation of neuronal morphology and synaptic transmission, we reasoned that the effects of BMEC were mediated by secreted factors. VEGF, as an endothelial and neuronal intrinsic factor, has been reported to regulate synaptic transmission (McCloskey et al., 2005; Kim et al., 2008; Huang et al., 2010; Tillo et al., 2012). Therefore, we assumed that VEGF involved in the endothelium’s effects. First we found that endothelial cells could release VEGF to the culture medium, and neuronal VEGF was increased under B-CM treatment (Figure 5A), suggesting that endothelial cells not only secrete VEGF, but also promote neuronal endogenous VEGF production. Thus we next explored the possibility that VEGF was responsible for the effect of endothelial cells on synaptic activity. To test this possibility, we treated neurons with B-CM and SU1498 (an inhibitor of Flk-1) and then recorded mEPSC. We observed that SU1498 repressed the enhancement of mEPSC frequency induced by B-CM, whereas SU1498 did not change mEPSC frequency of neurons cultured alone (Figures 5B,C).

To study the mechanism further, we next explored the signaling pathway of the endothelium’s effects and targeted p38 MAPK, which has been found to involve in the regulation of synaptic plasticity (Origlia et al., 2008; Liu et al., 2014). Indeed, we observed B-CM treatment resulted in significant p38 activation (Figure 5D), as shown by the robust increase in *p*-p38 expression. To assess the requirement of Flk-1 signaling in the induction of the activation of p38 MAPK signaling, we added SU1498 in the B-CM treated cultures. We found SU1498 inhibited B-CM-induced *p*-p38 upregulation, whereas SU1498 did not change *p*-p38 expression in neurons cultured alone (Figure 5E). The results highlight the importance of Flk-1 signaling in causing the activation of p38 MAPK signaling pathways, indicating that p38 MAPK signaling is the downstream pathways of Flk-1 signaling.

To determine if the p38 MAPK signaling mediated the effect of endothelial cells on synaptic activity, we treated neurons with B-CM in the presence of SB203580 (an inhibitor of p38 MAPK) and then recorded mEPSC. We observed that SB203580 inhibited the increase of mEPSC frequency induced by B-CM, whereas SB203580 did not change mEPSC frequency of neurons cultured alone (Figures 5F,G).

### SU1498 and SB203580 Inhibit the Increase of VGlut-1 and PSD95 Cluster Density of Cortical Neurons Induced by B-CM

**Figure 6:**
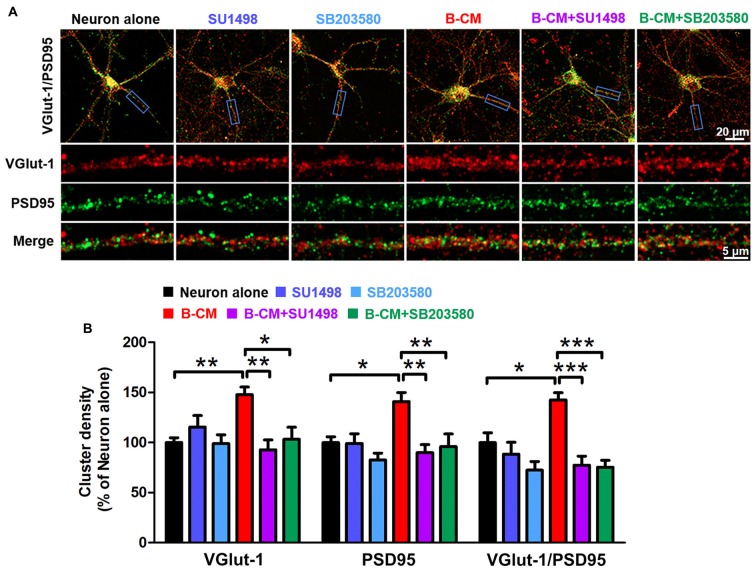
SU1498 and SB203580 inhibit the increase of VGlut-1 and PSD95 cluster density induced by B-CM. **(A)** Representative staining in clusters for VGlut-1 and PSD95 in cortical neurons cultured alone or treated with SU1498 (an inhibitor of Flk-1), SB203580 (an inhibitor of p38 MAPK), B-CM, B-CM+SU1498 or B-CM+SB203580. **(B)** Summary graphs showing the cluster density for VGlut-1, PSD95 and PSD95/VGlut-1 co-localization in the different treated neurons with values normalized to neuron alone group (*n* = 11 per condition. *p < 0.05; **p < 0.01; ***p < 0.001).

Based on the changes in synaptic transmission, synaptogenesis was examined in control and B-CM-treated neurons. After 12 DIV, cortical neurons were treated with B-CM for 2 days, and then the number of synapses was assessed by immunostaining for VGlut-1 (a presynaptic marker) and PSD95 (a postsynaptic marker) respectively. Synapses were determined as the co-localization of them. As shown in Figure 6, B-CM increased the number of excitatory synapses, as evidenced by more VGlut-1/PSD95 colocalized puncta in B-CM-treated neurons than those cultured alone. We also observed that B-CM treatment respectively increased the density of VGlut-1 and PSD95 clusters (Figure 6). Combined with the prior electrophysiological results, these findings suggest B-CM-induced an increase in mEPSC frequency is most likely attributable to an enhancement of presynaptic glutamate release through increasing VGlut-1-mediated glutamate vesicle transport.

To determine if VEGF/Flk-1/p38 MAPK signaling mediated the effect of endothelial cells on VGlut-1, PSD95 and synapse formation, we used the Flk-1 inhibitor, SU1498 and the p38 inhibitor, SB203580 respectively. The results showed that both SU1498 and SB203580 suppress the regulatory effect of B-CM on the synaptic protein expression (Figure 6), suggesting BMEC-secreted factors induce VGlut-1, PSD95 and synapse formation via VEGF/Flk-1/p38 MAPK signaling.

## Discussion

In this study, we observed that BMEC promoted neurite outgrowth, accelerated electrophysiological development as well as enhanced synapse formation and transmission via VEGF/Flk-1/p38 MAPK signaling. Our results strengthen the functional interdependence between the neurons and blood vessels during development. Additionally, we bring forward the idea that endothelial cells contribute significantly to the development and maintenance of neurovascular network in the developing CNS. From a clinical perspective, our study suggests that promoting angiogenesis may enhance synaptic connectivity providing a useful restorative intervention in neurodevelopmental or neurodegenerative diseases.

To investigate the regulation of BMEC on the maturation of neurons, we established a direct contact co-culture system with immature neurons and postnatal endothelial cells *in vitro* (Figure 1A). We found that BMEC could facilitate neuronal maturation with the following evidences: (i) BMEC promoted the spine formation of neurons and increased the cluster density of PSD95 in the later stage of *in vitro* development following it increased total length and complexity of neurites in the early stage (Figure 2), indicating that endothelial cells enhance morphological and functional maturation of neural dendrites, which account for the capacity of neurons to receive and integrate information from presynaptic (El-Husseini et al., 2000; Hlushchenko et al., 2016). Presynaptic marker VGlut1, which accumulates the glutamate into synaptic vesicles in glutamatergic axon terminals (Fremeau et al., 2004), was also increased by endothelial cells (Figure 6). Simultaneously, BMEC increased the cluster density of VGlut1/PSD95 co-localization indicating the number of synapses increased (Figure 6). Morphological analysis suggest that endothelial cells contribute to neuronal morphological maturation. (ii) At the electrophysiological level, we found passive membrane properties changed as development progresses and more neurons could produce APs under BMEC co-culture (Figures 3, 4). Moreover, BMEC co-culture enhanced the excitatory synaptic transmission of cortical neurons (Figure 4). Electrophysiological data indicate that endothelial cells accelerate neuronal functional maturation. Taken together, the cross-talk between BMEC and neurons facilitate both morphological and functional maturation of developing neurons.

To study whether endothelial effects on the neuronal development was caused by direct cell-cell contact or soluble factors released by endothelial cells, we used two culture systems including direct BMEC co-culture and B-CM treatment system. Our results show that effects of B-CM treatment on the neuronal development are similar to those of BMEC co-culture, indicating that BMEC regulate neuronal development via secreted factors but not in a direct cell-cell contact-dependent manner. In this study, we found that the endothelium-neuron interactions on the modulation of neuronal development was mediated by VEGF since SU1498, an inhibitor of VEGF receptor (Flk-1), could block B-CM-induced the enhancement of synapse formation (Figure 6) and excitatory synaptic transmission (Figure 5). These results further support previous data that administration of VEGF affects synaptic transmission via Flk-1 in hippocampal neurons (McCloskey et al., 2005; Kim et al., 2008; Huang et al., 2010). In our culture system, VEGF could be produced by both endothelial cells and neurons. We found that B-CM treatment upregulated neuronal endogenous VEGF expression (Figure 5A). Although previous finding that the endothelium-secreted factors such as IGF could induce neuronal VEGF (Huang et al., 2010) may partly explain our results, the detailed mechanism is still to be identified. The phenomena that endothelial cells could release VEGF and simultaneously upregulated neuronal VEGF expression raise the possibility that paracrine along with autocrine of VEGF for neurons may cooperatively regulate synapse formation and function. Further studies are necessary to investigate their exact function and interrelation in neuronal maturation.

It has been reported that VEGF principally acts as a paracrine factor for endothelial cells to stimulate angiogenesis in the developing brain (Ogunshola et al., 2000). Recent studies have revealed that VEGF is not solely an endothelial mediator, rather represent one of the major mediators in the neurovascular unit (NVU; Rosenstein and Krum, 2004; Mackenzie and Ruhrberg, 2012; Pan et al., 2017). VEGF could enhance neurogenesis and prolonged neurite outgrowth of newborn neurons in adult rat brains after ischemic stroke (Jin et al., 2002; Wang et al., 2009; Shen et al., 2016) beyond its effects on angiogenesis, which are in line with our study revealing the effects of VEGF on neural plasticity. Our findings provide novel aspects of VEGF in modulating neuronal maturation in NVU and extend the understanding of VEGF as a mediator in the endothelium-neuron interactions in the developing brain. Moreover, despite the potential side effects of VEGF, such as increased vascular permeability and formation of hemangioma, the data in this study illustrates that VEGF might be considered as a therapeutic target for neurodevelopmental or neurodegenerative disorders.

Next, we further explored the signaling mechanism of the endothelium’s effects and targeted p38 MAPK signaling pathway, which has been found to involve in the regulation of synaptic plasticity (Origlia et al., 2008; Liu et al., 2014). Here, these findings that inhibition of p38 MAPK significantly suppressed the endothelium-mediated the enhancement of synapse formation and transmission (Figures 5, 6) add to the evidence for a role for this kinase in the neural connectivity. Above all, the results indicate that endothelial cells promote synapse formation and excitatory synaptic transmission via activation of VEGF/Flk-1/p38 MAPK signaling pathway. In other words, VEGF/Flk-1/p38 MAPK signaling mediates the neurovascular interaction on the regulation of functional maturation in developing neurons.

BMEC form the NVU with neurons, astrocytes and extracellular matrix in the brains (Lo et al., 2003; Lok et al., 2007). It has been found that endothelial cells are able to regulate the neurogenesis (Leventhal et al., 1999; Shen et al., 2004; Gama Sosa et al., 2007) and protect neurons against injury (Guo et al., 2008; Wu et al., 2016). Our results provide novel aspects of endothelium-neuron interactions on the regulation of neural network. Of note, accumulating evidences suggest that glial cells modulate synapse formation and function in the developing CNS (Pfrieger and Barres, 1997; Ullian et al., 2001; Stogsdill and Eroglu, 2017). Here, we demonstrate a novel cell type in NVU, endothelial cells are capable of modulating synapse formation and function as well, indicating that glial and endothelial cells may act synergistically in regulating the synaptic transmission and maintaining synaptic stability in the brain.

**Figure 7:**
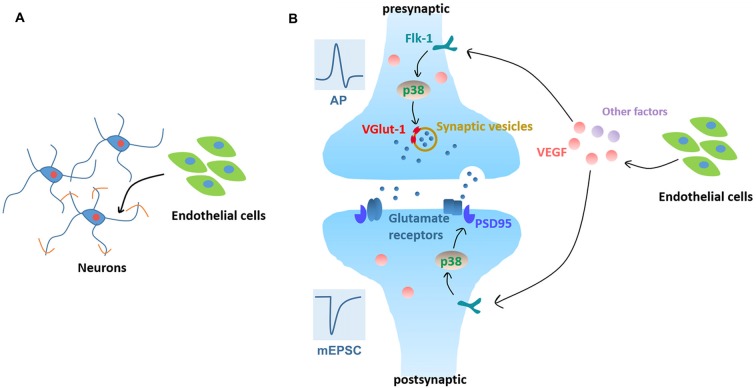
Working model. **(A)** Neurovascular interaction promotes neurite outgrowth in cortical neurons in the early stage of *in vitro* development. **(B)** Neurovascular interaction promotes functional maturation of neurons, as evidenced by facilitating APs production and increasing mEPSC frequency. Signaling pathways: neuronal Flk-1 engagement by VEGF results in the activation of p38 MAPK which induces presynaptic VGlut-1 and postsynaptic PSD95 and increases the excitatory synapse formation and transmission.

In summary, our data demonstrate that BMEC enhance neurite outgrowth, accelerate electrophysiological development and facilitate synapse formation and transmission. In addition, VEGF/Flk-1/p38 MAPK signaling mediates the enhancement of synapse formation and transmission induced by neurovascular interaction as summarized in Figure 7. We provide new insights in the development of brain, both in terms of neural circuit maturation and neurovascular interaction development.

## Ethics Statement

This study was carried out in accordance with the National Institutes of Health Guide for the Care and Use of Laboratory Animals. The protocols were approved by the Ethics Committees of Experimental Research of the Shanghai Medical College of the Fudan University.

## Author Contributions

K-WW and F-YS designed experiments, analyzed data and wrote the manuscript; K-WW, J-LM, Z-WK, QL, L-LL, and YL performed experiments and collected data. All authors revised the manuscript.

## Conflict of Interest Statement

The authors declare that the research was conducted in the absence of any commercial or financial relationships that could be construed as a potential conflict of interest.
