# Supplementary figures and images for: Neurovascular Interaction Promotes the Morphological and Functional Maturation of Cortical Neurons
Source: Front Cell Neurosci. 2017 Sep 15;11:290. doi: 10.3389/fncel.2017.00290 (PMC5605567; doi:10.3389/fncel.2017.00290)

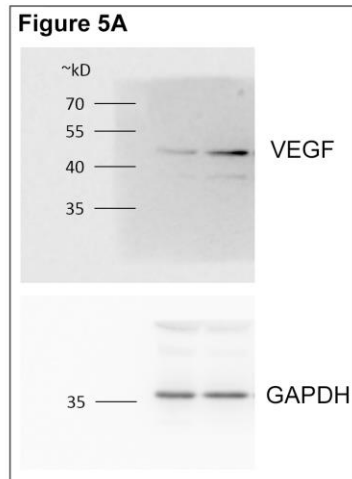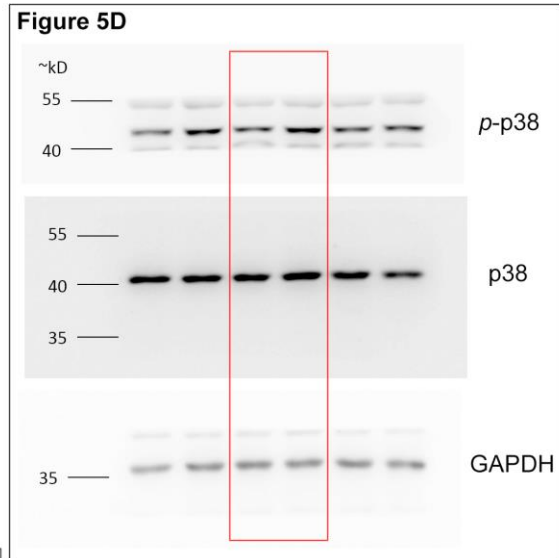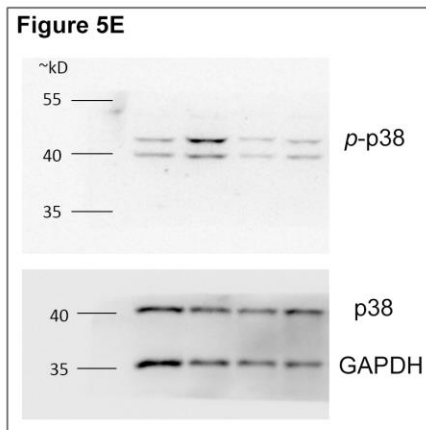

**Supplementary Figure 1. The original images of blots in Figure 5.**

Supplement: Supplementary file 1 [file Image_1.pdf]
